# A retrospective study of 18 children with subcutaneous panniculitis-like T-cell lymphoma: multidrug combination chemotherapy or immunomodulatory therapy?

**DOI:** 10.1186/s13023-022-02575-4

**Published:** 2022-12-12

**Authors:** Yanlong Duan, Huixia Gao, Chunju Zhou, Ling Jin, Jing Yang, Shuang Huang, Meng Zhang, Yonghong Zhang, Tianyou Wang

**Affiliations:** 1 Medical Oncology Department, Pediatric Oncology Center, Beijing Children’s Hospital, National Center for Children’s Health, Beijing Key Laboratory of Pediatric Hematology Oncology, Key Laboratory of Major Diseases in Children, Ministry of Education, National Key Discipline of Pediatrics, Capital Medical University, Beijing, 100045 People’s Republic of China; 2 Pathology Department, National Center for Children’s Health, Beijing Children’s Hospital, Capital Medical University, Beijing, 100045 People’s Republic of China; 3 Hematology Center, Beijing Children’s Hospital, Capital Medical University, Nanlishi Road No. 56, Xicheng District, Beijing, 100045 People’s Republic of China

**Keywords:** Subcutaneous panniculitis T-cell lymphoma, Clinicopathological characteristics, Immunomodulator agents, Chemotherapy, Children

## Abstract

**Background:**

Subcutaneous panniculitis T-cell lymphoma (SPTCL) is a rare, cytotoxic T-cell lymphoma with which some patients have accompanying hemophagocytic syndrome (HPS). There is currently no standard treatment regimen. In the past, the most commonly used treatment was multidrug chemotherapy. In contrast, numerous case reports or small series suggest that immunosuppressive drugs could also be effective for some patients. Since this NHL subtype is extremely rare in children and adolescents, to improve the understanding of this disease and standardize its rational treatment, we retrospectively summarized the treatment regimens of 18 pathologically diagnosed children with SPTCL to compare the clinical efficacy of multidrug chemotherapy and immunomodulatory therapy.

**Results:**

The median age of onset was 11.1 years. Painless subcutaneous nodules or skin patchy lesions were found in all patients, most commonly involving the lower extremities and/or trunk. Before January 1, 2019, the treatment was mainly chemotherapy, and 10 patients were initially treated with chemotherapy, among whom was one patient who progressed during initial treatment, was voluntarily discharged and was subsequently lost to follow-up, one patient who died of disease progression, and the remaining 8 patients who all achieved sustained remission, with a complete remission (CR) rate of 80% (8/10). Corticosteroids combined with cyclosporine A or ruxolitinib were the most common initial immunosuppressive agents at our center after January 1, 2019 and had a CR rate of 71.4% (5/7). In addition, 1 patient achieved partial remission (PR) during follow-up, and one had autologous hematopoietic stem cell transplantation (AHSCT) after 4 months of drug withdrawal. There were 7 patients (38.9%, one case in chemotherapy group and six cases in immunotherapy group) with HPS and 4/5 screened patients (80%) with positive *HAVCR2* gene mutations. The median follow-up was 17 months.

**Conclusion:**

The prognosis of SPTCL is relatively good. Previous multi-drug and long-term chemotherapy treatment has clear efficacy, and recent immunomodulatory therapy as pre-chemotherapy therapy can also benefit patients.

## Introduction

Subcutaneous panniculitis T-cell lymphoma (SPTCL) is a rare and highly heterogeneous cytotoxic T-cell lymphoma that accounts for less than 1% of all peripheral T-cell lymphomas [1]. SPTCL mainly accumulates subcutaneous adipose tissue. Some SPTCL patients also have hemophagocytic syndrome (HPS), which progresses rapidly and has a relatively poor prognosis [2]. The diagnostic criteria of SPTCL have been described and clearly defined, yet there is currently no standard treatment regimen. In 2008, the European Organization for Research and Treatment of Cancer (EORTC) conducted a major study highlighting the favorable prognosis of SPTCL and the adverse impact of HPS on survival [3]. In that study, most patients were treated with polychemotherapy, while approximately one-third received immunosuppressive drugs. However, the respective efficacy of these treatments was not evaluated. In the past, the most commonly used treatment was multidrug chemotherapy, which had CR rates ranging from 0 to 67% reported in the literature [4–6]. In contrast, numerous case reports or small series suggest that immunosuppressive drugs could also be effective [7–9]. The French Study Group of Cutaneous Lymphoma (GFELC) favors a conservative approach, with immunosuppressive drugs as the first-line treatment, continuing for as long as the patient’s health allows. The EORTC has also recommended immunosuppressive therapy as a first-line treatment for SPTCL patients without HPS [3].

Due to the extreme rarity of such NHL subtypes in childhood, prospective therapy trials have posed significant challenges and, therefore, are lacking. To date, most reports about SPTCL in children both in China and elsewhere have been individual cases. The 18 cases of SPTCL that were confirmed by a pathology review at our single-center are the largest number of childhood cases reported, thus far. To improve the understanding of this disease and standardize rational treatment, we conducted this retrospective study to compare the clinical efficacy of multidrug chemotherapy to immunomodulatory therapy.

## Data and methods

### Patients and clinical data

The clinical data of 18 SPTCL patients admitted from January 1, 2010, to December 31, 2021 were collected at Beijing Children's Hospital (affiliated with Capital Medical University). All diagnoses were confirmed by a pathological biopsy of the skin or subcutaneous nodules, and some tissue samples were sent to the Pathology Department of Friendship Hospital (affiliated with Capital Medical University) for verification. One or more biopsy samples were collected for each case. The following panel of antibodies was used: CD2, CD3, CD4, CD5, CD8, CD56, CD68, TIA-1, Granzyme B, Ki-67 and βF1 (Santa Cruz Biotechnology, Inc., Dallas, TX, Sweden, USA). Epstein‒Barr virus-encoded RNA (EBER) in situ hybridization using EBER oligonucleotides was performed on fixed sections using a Dako hybridization kit (Dako, Copenhagen, Denmark). Routine blood tests, liver and kidney function, and lactate dehydrogenase (LDH) tests were performed on all patients before treatment. We retrieved clinical data, such as patients’ clinical characteristics, treatment, and outcome, from their medical records. The Institutional Review Board of Beijing Children’s Hospital approved this study.

### Diagnosis criteria

SPTCL was diagnosed according to the WHO criteria [10]. HPS was defined according to the HLH-2004 criteria [11]. The toxic and side effects of drugs were determined by reference to CTCAE4.0 [12], which included hematological and non-hematological indicators.

### Follow-up and efficacy evaluation

Responses to treatment were classified as CR, partial remission (PR), or stable disease (SD) based on the Lymphoma International Workshop criteria in 2007 [13]. The duration of CR(DOR) was defined as the time between the date of CR and the date of relapse or last follow-up. Patients were followed in the outpatient clinic and by telephone until December 31, 2021, the date was regarded as the endpoint of our observation. The date of death or loss to follow-up was also regarded as the follow-up endpoint.

### Statistical analysis

Percentages were compared using Fisher’s exact test. All statistical tests were two-tailed, with a significance level of 0.05. Analyses were performed using SPSS 19.0 statistical software.

## Results

### Clinical description

The median age of the 18 patients was 11.1 years (0.52–14.7), and 3 patients were under 1 year old, accounting for approximately 16.7% of the sample. There were 11 males and 7 females, with a male-to-female ratio of 1.5. Ten of the 18 patients received chemotherapy (six patients received NKT-like chemotherapy, and four received ALL-like chemotherapy), seven patients received immunomodulator drugs (corticosteroids combined with cyclosporine A or ruxolitinib), and one was followed up in the outpatient department after skin mass resection. The first course of chemotherapy lasted between 4 and 6 courses. The chemotherapy regimens and compositions are shown in Table 1. The initial symptoms of the patients were mainly single or multiple subcutaneous nodules or skin patchy lesions, with little infiltration outside the skin, and some patients had spontaneous regression in the early stage of the disease. Typical lesions were more frequently found on the limbs and/or trunk (12 cases) but less on the head, neck and face (6 cases). In addition, 11 patients (61.1%) had B-symptoms (fever, asthenia, and weight loss). An abnormal cell count was observed in most patients, with a normal low leukocyte count (3.4 ± 1.4) and high lymphocyte count (49.0 ± 11.7). HPS was suspected in 7 patients (38.9%) and confirmed by bone marrow biopsy with a phagocytic phenomenon and either hyperferritinemia or hypertriglyceridemia. In summary, we did not observe any significant differences in clinical presentation between the two groups, except for the incidence of HPS (*P* = 0.004), and HPS mainly occurred in the immunomodulator drug group, as shown in Table2. In addition, the comparison of adverse reactions of the two groups showed that the multi-drug chemotherapy group accounted for a larger proportion(Table 3).Five patients were screened for the *HAVCR2* gene mutation in T-cell immunoglobulin domain and mucin domain 3 (TIM-3) lineages, and four (80%) were positive: one patient with early hematophagy was undergoing cyclosporine maintenance therapy after PR with chemotherapy, one relapsed 4 months after rucotinib withdrawal, and two with sustained CR who are receiving modulated immunoregulation therapy are still being followed. Another 3 patients were screened for genetic predisposition of genes associated with blood tumors or immune deficiency diseases, as shown in Tables 4 and 5.

**Table 1:** Chemotherapy regimens and components for 18 cases of SPTCL

Regimen	Agents
NKT-like	SMILE: steroids, methotrexate, ifosfamide, L-asparaginase, etoposide
ALL-like	VDLD: vincristine, daunorubicin, L-asparaginase, dexamethasone CAM: cyclophosphamide, cytarabine,6-mercaptopurine MTX + 6MP + VD: methotrexate + 6-mercaptopurine + vincristine, daunorubicin HD-MTX: high dose of methotrexate
HLH-04 protocol Immunosuppressant Intrathecal	Prednisone, Cyclosporine A Ruxolitinib, tacrolimus Dexamethasone, cytarabine, methotrexate

*SPTCL* Subcutaneous panniculitis T-cell lymphoma, *NKT- like* Natural killer T cell-like, *ALL-like* Acute lymphoblastic leukemia-like, *HLH-04* Hemophagocytic lymphohistiocytosis-04 protocol

**Table 2:** Treatment and outcome of patients with SPTCL

	Group 1	Group 2	*p*
First-line treatment, n (%)	7(41.2)	10(58.8)	
Age, years, median	10.8	8.7	0.38
Sex, n1/n2	4/3	6/4	0.99
B symptom, n (%)	5/7(71.4)	6/10(60.0)	0.99
Haemophagocytic syndrome, n (%)	6/7(85.7)	1/10(10.0)	0.004
Complete remission, n (%)	6/7(85.7)	8/10(80.0)	0.99
Deceased, n (%)	0/7(0)	1/10(10.0)	0.99

Group 1: Immunomodulator therapy; Group 2: Polychemotherapy; n1 = Male; n2 = Female

**Table 3:** Comparison of adverse reactions between the two groups [n (%)]

Group	Numbers of cases	Neutropenia	Anemia	Thrombocytopenia	Pulmonary infection	Cardiotoxicity	Vomiting
Group 1	7	2[28.6]	4[57.1]	3[42.6]	2[28.6]	0[0]	1[14.3]
Group 2	10	8[80.0]	7[70.0]	4[40.0]	6[60.0]	1[10.0]	5[50.0]
χ2		4.496	0.298	0.014	1.633	0.744	2.300
*P*		0.058	0.644	> 0.999	0.335	> 0.999	0.304

Fisher's Exact test was used, *P* > 0.05

Group 1: Immunomodulator therapy; Group 2: Polychemotherapy

**Table 4:** Clinical characteristics, treatment and prognosis of 18 SPTCL cases

Age/sex	Extent of cutaneous lesions	B symptoms	HPS	Chemotherapy regimen	Relapse	Clinical stage	Prognosis
12y/M(P1)	Multiple, extremities, face	No	No	NKT-like, IT	No	T1N1M0,IV	CR (76 months)
12y/F(P2)	Single, extremities	Yes	No	NKT-like, IT	No	T1N0M0	CR (17 months)
10 m/M(P3)	Single, extremities	No	No	NKT-like, IT	No	T1N0M0	CR (17 months)
13y/F(P4)	Single, extremities	Yes	Yes	NKT-like	Yes	T1N1M0,IV	CR (26 months), relapse
11y/M(P5)	Single, trunk	Yes	No	ALL-like, IT	No	T3N0M0	CR (58 months)
5 m/M(P6)	Multiple, extremities, face	No	No	NKT-like, IT	No	T1N0M0	CR (21 months)
2y/F(P7)	Multiple, extremities, trunk	Yes	No	NKT-like, IT	No	T1N1M0,IV	CR (34 months)
10y/F(P8)	Single, extremities	Yes	No	ALL-like, IT	No	T1N1M0,IV	CR (44 months)
11y/F(P9)	Multiple, extremities, trunk	No	Yes	HLA-04, Ruxo	No	T1N1MO,IV	CR (14 months)
12y/F(P10)	Single, trunk	Yes	Yes	HLA-04	No	T1N0M0	PR, Continue treatment
10y/M(P11)	Single, perineum	Yes	Yes	Glucocorticoid, Ruxo, tacrolimus	No	T1N0M0	CR (9 months)
12y/M(P12)	Single, face	Yes	No	ALL-like, IT	No	T1N1M0,IV	CR (74 months)
9y/F(P13)	Single, extremities	No	No	Prednisone, hydroxychloroquine	No	T3NxM1IVB	Disappeared spontaneously
14y/M(P14)	Multiple, extremities, face	Yes	Yes	HLA-04	No	T1N0M0	CR (27 months)
12y/M(P15)	Multiple, extremities, face	Yes	Yes	Glucocorticoid, Ruxo	No	T1N0M0	CR (10 months)
5y/M(P16)	Single, trunk	Yes	Yes	Glucocorticoid, Ruxo	Yes	T1N0M0	ASCT, follow-up
11 m/M(P17)	Multiple, neck, face	No	No	Excision only	No	T1N0M0	Follow-up (6 months)
11y/M(P18)	Multiple, extremities, trunk	No	No	ALL-like	No	T1N0M0	Death

*F* Female, *M* Male, B symptoms: fever, night sweats, weight loss; NKT-like = SMILE: steroids, methotrexate, ifosfamide, L-asparaginase, etoposide; ALL-like = VDLD + CAM + MTX + 6MP + VD + HD-MTX; IT = Intrathecal(Dexamethasone, cytarabine, methotrexate); HLA-04: Prednisone, Cyclosporine A;ASCT: Autologous Stem Cell Transplantation; Ruxo: Ruxolitinib; T = Tumor; N = Node; M = Metastases; CR: Complete remission PR: Partial remission

**Table 5:** Features of gene mutation in 8 SPTCL cases

Patient	*HAVCR2*(TIM-3)	Germline status	Blood and immune system disease genetic susceptibility gene testing
P9	Y82C	Homozygous	–
P10	Y82C	Homozygous	–
P11	Y82C	Homozygous	–
P16	Y82C	Homozygous	–
P17	Negative	–	–
P2	–	Non-Synonymous	RAD9A:Chr11: NM_004584, exon8:c.A716G p.H239R,VF:44.00%
P6	–	Non-Synonymous	KLF1:Chr19:NM_006563:exon2:c.T544C,p.F182L,VF:41.18%;GALT:Chr9,NM_000155:exon10:c.A940G,p.N314D,VF:46.67%; GALC:Chr14:NM_000153:exon16:c.T1901C,p.L634S,,VF:36.73%
P7	–	Non-Synonymous	CARD9:Chr9-139,265,289:NM_052813:exon5:c.627 + 4C > T, Chronic mucocutaneous candidiasis type 2; CYBB:ChrX-37665739:NM_000397:exon11:c.1414G > A,p.G472S,Chronic granuloma, immunodeficiency type 34; LRBA:Chr4-151,935,743:NM_006726:exon2:c.52G > A,p.G18R,Immunodeficiency type 8 with autoimmune disease; LIG4:Chr13-108,861,645:NM_002312:exon2:c.1972A > G,p.I658V,LIG4 syndrome

*VF* Variable frequency

### Histological and immunohistochemical analysis

Histopathologically, all diagnostic skin biopsy specimens showed dense subcutaneous infiltrates with a pattern of lobular panniculitis. Atypical lymphocytes were generally small to medium in size with obvious nuclear atypia. Neoplastic T-lymphocytes [CD2^+^(16/18), CD3^+^(18/18), CD4^−^(16/18), CD5^+^(14/18), CD8^+^(18/18), CD56^−^(17/18), CD68^+^(17/18), TCR-βF1^+^(15/18)] usually showed a TIA1 cytotoxic granule associated RNA binding protein 1–positive phenotype with a high proliferation rate (Table 6). EBER in situ hybridization test results were negative.

**Table 6:** Clinical and pathological features of 18 SPTCL cases

Sample	P1	P2	P3	P4	P5	P6	P7	P8	P9	P10	P11	P12	P13	P14	P15	P16	P17	P18
Ki-67(%)	60	30	65	45	50	65	20	40	40	30	25	20	50	35	65	80	40	30–50
CD2	+	+	+	+	+	+	+	+	+	+	+	+	+	+	−	+	−	+
CD3	+	+	+	+	+	+	+	+	+	−	+	+	+	+	+	+	+	+
CD4	−	−	−	−	−	−	−	−	−	−	−	−	−	−	−	+	+	−
CD5	+	+	+	−	+	+	+	+	+	+	+	+	+	−	−	+	−	+
CD8	+	+	+	+	+	+	+	+	+	+	+	+	+	+	+	+	+	+
CD30	−	−	−	−	−	−	−	−	−	−	−	−	−	−	−	−	−	−
CD56	−	−	−	−	−	−	−	−	−	−	−	−	−	−	−	−	−	+
CD68	+	+	+	+	+	−	+	+	+	+	+	+	+	+	+	+	+	+
TIA-1	+	+	+	+	+	+	+	+	+	+	+	+	+	+	+	−	+	+
Gram-B	+	+	+	+	+	+	+	+	+	+	+	+	+	+	+	−	+	+
TCR-βF1	−	+	+	+	+	+	+	−	−	+	+	+	+	+	+	+	+	+
EBER	−	−	−	−	−	−	−	−	−	−	−	−	−	−	−	−	−	−
WBC	2.9	4.6	5.1	1.4	3.3	2.9	6.9	3.6	3.1	3.8	2.6	3.6	2.9	0.9	4.5	4.5	2.3	4.8
LC(%)	45.1	37.2	44.2	66.9	47.9	75.7	51.2	52.6	53.3	26.4	57.7	56.7	54.6	50.1	33.8	41.2	43.1	40.3
LDH	2011	316	296	679	1720	593	419	1310	1452	1249	589	103	299	995	1269	2087	269	364
EBV-DNA	−	−	−	−	−	−	−	−	−	−	−	−	−	−	−	−	−	−
ENA-Abs	−	−	−	−	−	−	−	−	*	−	−	−	**	−	−	−	−	−
TCR- rearrangement	NA	+	+	NA	NA	NA	NA	NA	NA	NA	NA	NA	−	+	+	+	+	+

*TIA-1* T-cell intracellular antigen, *EBER* Epstein-Barr-virus encoded small nuclear RNA, *TCR* T-cell receptor, *LC *Lymphocyte, *LDH *Lactate dehydrogenase, *ENA-Abs* ENA-Antibodies; *:antinuclear antibody1:80,anti-RNP antibody weakly positive, anti-SSA antibody positive, anti-RO-52 antibody positive, anti-dsDNA/Sm antibody negative, lupus anticoagulant negative;**:anti-extractable nuclear antigen antibody weakly positive, JO-1 weakly positive, anti-rRNP antigen antibody weakly positive; *NA* Not available

### Treatment and outcome

The first-line treatments in this study were either conventional, multidrug chemotherapy (55.5%) or immunomodulatory drugs (38.9%), except for one patient (5.6%) who was treated by surgical intervention. Chemotherapy, the main treatment before January 1, 2019, was the initial treatment for 10 cases, including 8 cases treated with an intrathecal injection. Since the toxicity of chemotherapy regimens mainly involves bone marrow suppression, all patients received granulocyte-stimulating factor support therapy after receiving polychemotherapy. After January 1, 2019, immunomodulatory therapy or observational follow-up was the main regimen. Seven patients were treated with immunomodulator agents, principally glucocorticoids combined with cyclosporine A or ruxolitinib. One patient was followed after surgical resection of the lesion. By the end of follow-up, there were 14 patients (77.8%) with persistent CR. The remaining 4 patients were as follows: One patient died of septic shock after receiving ALL-like chemotherapy, one patient with NTK-like chemotherapy relapsed and was discharged and, ultimately, lost to follow-up after 1 year of drug withdrawal, one patient received autologous hematopoietic stem cell transplantation (AHSCT) after 4 months of lucotinib immunomodulator treatment, and one patient achieved PR to current immunomodulator treatment during the last follow-up. Among the 7 patients with HPS, one was treated with chemotherapy and the remaining 6 patients were all received immunotherapy, of these 6 patients: one developed a recurrent mass 4 months after the withdrawal of ruxolitinib immunotherapy and was prepared for AHSCT, one achieved PR, and the remaining 4 patients were in all a persistent CR state. The median follow-up was 17 months (6–76 months).

## Discussion

This single-center, retrospective study of 18 pathologically confirmed cases of SPTCL, treated with either polychemotherapy or immunomodulator agents is the largest series of pediatric cases reported to date. The results showed that 18 patients with SPTCL were diagnosed in nearly 2000 Non-Hodgkin's Lymphoma admitted to our single center in 10 years. Of these 18 patients, except one died and two relapsed after treatment, the remaining 15 patients remained in event-free survival state up to the follow-up date. In the past, the efficacy of multidrug and long-term chemotherapy treatment was clear, and recent studies indicate that immunomodulatory treatment can also benefit patients as a first-line treatment before multidrug chemotherapy.

The clinical manifestations of SPTCL are highly heterogeneous. In clinical practice, based on the experience of our single center, we have observed spontaneous remission in younger children (onset age less than 1 year), while older children were often complicated with HPS. The SPTCL diagnosis is based on pathological examination, and immunophenotype and T-cell receptor gene (TCR) rearrangement also have important diagnostic value. The histopathological features and immunohistochemical results in our study were similar to those of other studies reported in the literature (see Table 6 for details).

SPTCL is exceedingly rare in clinical practice; therefore, our understanding of this disease is not comprehensive. The exact etiology and pathogenesis of SPTCL are still unclear. In the past, some scholars thought that the Epstein‒Barr virus infection might be associated with its occurrence [14], but an increasing number of studies have shown that the disease and the Epstein‒Barr virus do not have an obvious correlation [15]. The EB virus infection was not detected by EBV-DNA and EBER in situ hybridization in our 18 children. Huppmann [16] highlighted that pediatric SPTCL is frequently associated with autoimmune diseases or genetic/developmental abnormalities and proposed that SPTCL might be the result of clonal lymphocyte proliferation caused by the dysregulation of the response after the recognition of autoantigens or infectious antigens by T cells. In this study, the antinuclear antibody spectrum of two children was partially positive, but since there is no specific index to diagnose autoimmune diseases, it was speculated that these conditions could be attributed to susceptibility. Potential hematological tumor or immune deficiency susceptibility gene variants were detected and reported in 3 patients who underwent gene testing for genetic susceptibility (see Table 5). This also suggests that SPTCL or immune-related diseases or tumors may have a common cause. Recent studies on the functional impairment due to germline *HAVCR2* mutations of TIM-3 in some SPTCL patients have provided some clues to understanding the underlying pathogenesis of SPTCL [17, 18]. TIM-3 is a negative immune checkpoint expressed in T-cell subsets, and it regulates T-cell function. Its functional impairment can promote the activation of T lymphocytes and macrophages and significantly increase the level of proinflammatory cytokines. As a negative immune checkpoint expressed in T-cell subsets, its functional impairment ultimately leads to the activation of T lymphocytes and macrophages as well as increased levels of proinflammatory cytokines [18, 19]. TIM-3 mutant SPTCL, however, is associated with hemophagocytic lymphohistiocytosis—a refractory and severe disease course. This also suggests that SPTCL patients with TIM-3 dysfunction may benefit from immunomodulatory therapy [19, 20]. Gabrielle Sonigo et al. [21] evaluated *HAVCR2* mutations in 53 of 70 SPTCL patients from 19 medical centers in France from 2000 to 2019 and found that *HAVCR2* mutations were present in 25% of patients, which is a figure much lower than the 85% reported in Asia. It should be emphasized that all four patients with *HAVCR2* gene mutations had HPS. Therefore, we recommend that patients with HPS should be tested for *HAVCR2* mutation status [22] to identify those patients requiring intensive treatment.

SPTCL is considered as having a good prognosis, although reported overall survival varies between 64 and 82% [5, 23, 24]. However, HPS occurs in 15–20% of patients, and the 5-year survival rate of these patients is only 46% [25]. There is no clear consensus about the best therapeutic approach. In practice, the most commonly used treatment is polychemotherapy, which has CR rates ranging from 0 to 67% of reported cases. This variability could reflect differences in disease severity among studies, as well as differences in therapeutic strategies. In recent years, corticosteroids in combination with other immunomodulator drugs were the preferred treatment of a "dimensional-lowering strike" tried with the understanding of the disease. Immunosuppressants (such as cyclosporine A) have been reported to achieve good results in the treatment of SPTCL patients without HPS [5–7, 26]. The European Organization for Research and Treatment of Cancer has also recommended immunosuppressive therapy as a first-line treatment for SPTCL patients without HPS [1].

Considering the increasing number of cases achieving CR with immunomodulator drugs, we compared two strategies for the first-line treatment of patients admitted over the last 10 years. Although HPS has been described as a major prognostic factor and has been associated with lower 5-year overall survival [1]. However, we were unable to identify HPS as a factor affecting survival prognosis due to the overall low mortality of SPTCL. This difference was related to our small number of study cases. However, according to the previous literature, patients with SPTCL combined with HPS are a treatment challenge. Cyclosporine was first used in adults with refractory SPTCL (CHOP failed, at least 1 salvage treatment) by Rojnuckarin et al. [27]. Four patients showed rapid improvement within weeks of starting cyclosporine (4 mg/kg/d), of whom three achieved persistent CR in that case series. The French group reported 27 cases of SPTCL patients (37% with HPS) with completely different treatment methods [28]; 69.5% of their patients received immunosuppressive drugs (Group 1), and 30.5% received polychemotherapy (Group 2). The CR rate was significantly better in the first group than in the second, with progression occurring less commonly [81.2% vs. 28.5% (*P* = 0.025) and 6.2% vs. 42.8% (*P* = 0.067), respectively]. The results suggest that the immunomodulator strategy for SPTCL may be effective as first-line therapy. In our study, as of the end of follow-up, CR rates in the chemotherapy group and immunomodulator group were 80.0% (8/10) versus 71.4% (5/7), respectively, while the HPS co-occurring rates in the two groups were 10% (1/10) versus 85.7% (6/7), respectively. Although the benefits of immunomodulator drug therapy are uncertain due to the relatively limited number of patients and short follow-up periods, it appears that patients are more likely to benefit from immunomodulator therapy, based on both the current understanding of the pathogenesis and the additional risks of chemotherapy. There is no evidence that all patients with SPTCL should be treated with chemotherapy or more aggressive treatment. Long-term follow-up has shown that patients with recurrent SPTCL had better efficacy after a continuous application of cyclosporine A [29]. It is suggested that patients who have poor efficacy on chemotherapy regimens can be combined with cyclosporine A as soon as possible to achieve better efficacy. Altogether, the findings from this study indicate that immunomodulatory treatment can also benefit SPTCL patients as a first-line treatment before multidrug chemotherapy, although some may benefit from polychemotherapy or HSCT in case of treatment failure.

## Conclusion

We reviewed the complete clinical therapeutic information of 18 children with SPTCL in our single center. Our results showed that previous multi-drug and long-term chemotherapy treatment has clear efficacy, and our recent studies indicate that immunomodulatory treatment as pre-chemotherapy therapy can also benefit patients and improved *HAVCR2* mutation testing to identify patients requiring intensive treatment for HPS.

## Data Availability

Anonymized data can be made available upon reasonable request.

## Abbreviations

SPTCL: Subcutaneous panniculitis T-cell lymphoma
HPS: Hemophagocytic syndrome
CR: Complete remission
AHSCT: Autologous hematopoietic stem cell transplantation
EORTC: European Organization for Research and Treatment of Cancer
GFECL: French Study Group of Cutaneous Lymphoma
PR: Partial remission
SD: Stable disease
TCR: T-cell receptor gene
